# New Insight into AuNP Applications in Tumour Treatment and Cosmetics through Wavy Annuli at the Nanoscale

**DOI:** 10.1038/s41598-018-36459-0

**Published:** 2019-01-22

**Authors:** Sara I. Abdelsalam, M. M. Bhatti

**Affiliations:** 1 Basic Science, Faculty of Engineering, The British University in Egypt, Al-Shorouk City, Cairo 11837 Egypt; 2 Instituto de Matemáticas - Juriquilla, Universidad Nacional Autónoma de México, Blvd. Juriquilla 3001, Querétaro, 76230 Mexico; 3 Shanghai Institute of Applied Mathematics and Mechanics, Shanghai University, Shanghai, 200072 China

## Abstract

The purpose of this study is to probe the peristaltic propulsion of a non-Newtonian fluid model with suspended gold nanoparticles. The base fluid is considered to simulate blood using the Carreau fluid model. We model a small annulus as a tube with a peristaltic wave containing a clot propagating towards the tube wall. An external variable magnetic field is also considered in the governing flow. An approximation for long wavelengths and small Reynolds numbers is employed to formulate the governing flow problem. The resulting nonlinear equations are solved using a perturbation scheme. Series solutions are obtained for the velocity profile, temperature profile, pressure rise and streamlines. The results indicate an enhancement in the temperature profile that can be utilized in eradicating tumour cells.

## Introduction

Gold nanoparticles (AuNPs) have been widely applied since their use in treating cancer patients was legislated by the Egyptian Health Ministry^1^. Although it may appear that this type of treatment is costly, it has been clinically proven that treatment with gold is less costly than chemotherapy; overall, the treatment cost is less than the price of a wedding ring!^2^ State-of-the-art treatments have been developed to improve the efficacy and reduce the side effects of other cancer therapies. AuNPs range in size from 10^−8^ to 10^−9^ m; because AuNPs are very small and can easily penetrate throughout the desired organ, they are currently used in cancer treatment. AuNPs can also link drugs and proteins to each other, which increases the ability of cancer treatment to eradicate tumour cells both *in vitro* and *in vivo*^3^. The procedure for destroying a malignant tumour primarily depends upon concentrating laser pulses with a specific wavelength on AuNPs, which in return can destroy cancer tissues^4,5^. It has been shown that when a nanosecond laser beam at the appropriate wavelength is directed on cells *in vitro*, ninety-five percent of cells treated with AuNPs are eradicated^2^.

Researchers have suggested that blood clots in an abdominal vein may be an indicator of undiagnosed cancer^6^. Furthermore, according to the Cancer Research Institute in the UK, cancer patients may have a higher risk of developing blood clots; this condition is known as a deep vein thrombosis (DVT), which has been studied by Cieslik *et al*.^7^. Finally, patients with skin cancer can suffer from abnormal blood clotting (thrombocytopaenia) after chemotherapy, as seen in many studies, including one at the Milton S. Hershey Medical Center in the USA^8^.

Despite the hype regarding motion through rigid boundaries in the last decade, peristaltic transport is a cutting-edge field with numerous applications in biomedicine, physiology, chemistry, nuclear reactors, and more^9–13^. However, there have been few studies incorporating NPs with peristalsis through several conduits. The concept of peristaltic pumps through wavy annuli has recently been prominence in many biological applications such as catheterized arteries and endoscopy. With the injection of an indwelling catheter inside a human or animal, organs can adjust the flow and modulate the pressure distribution. An endoscope can further inspect hollow organs, where a straight line-in-sight observation has previously been unattainable. Considering the significance of research about using peristaltic pumps to move physiological fluids with NPs through annuli, various investigators have studied peristalsis through an annulus with natural convection. Hatami *et al*.^14^ investigated the effect of a uniform transverse magnetic field in the porous medium of a hollow vessel containing blood, as a third-grade fluid, along with AuNPs. The authors used numerical and analytical approaches, in which their results were found in accurate agreement. Later, Nadeem *et al*.^15^ used the homotopy perturbation technique to investigate the peristalsis of an incompressible nanofluid in the porous region of a vertical annulus; equations were derived and solved with a low Reynolds number and long wavelength approximation. The researchers concluded that the peristaltic pumping rate increases as the local NP Grashof number is increased. Abdellateef *et al*.^16^ investigated the peristaltic transport of a Newtonian nanofluid in an inclined annulus with heat transfer occurring at nonslip inner and outer boundaries. Five distinct nanofluids were investigated by Hayat *et al*.^17^ in an asymmetric channel in the presence of a magnetic field; the authors demonstrated that the nanofluid decelerates as the magnetic field strength increases. Finally, Mekheimer *et al*.^18^ studied AuNPs using a non-Newtonian fluid model of peristalsis in a gap between two coaxial tubes.

The heating effect is specifically strong for metal NPs due to the number of valence electrons resulting in metallic bonding. The mechanism of using heated AuNPs to treat tumour cells has been studied by many researchers^19^. In this approach, NPs are bound to cancer cells using biomolecular linkers, and the thermal energy originating from optically stimulated NPs destroys the cancer cells. In addition, many investigators have studied the peristalsis of non-Newtonian fluids through coaxial tubes with heat transfer since the transmission of thermal energy across a well-defined boundary in thermodynamic systems with peristalsis is significant in biomedical systems. Hayat *et al*.^17^ investigated the peristaltic motion of water-based NFs for two thermal conductivity models. The authors concluded that the NP volume fraction increases with an increasing heat transfer rate. Further, a water-based NF with carbon NPs was studied by Akbar^20^ in an endoscope with thermal effects. It was concluded that the temperature of single-walled carbon nanotubes is greater than that of multi-walled carbon nanotubes. For more noteworthy biological applications of AuNPs, the reader is referred to^21–24^.

Often, the heating effect in metallic NPs is amplified in the presence of electromagnetic radiation^19^. Moreover, the combination of magnetic fields with peristaltic flows has significant applications in biomedical engineering problems^11,15–18,20,23–28^. Abdelsalam and Vafai^29^ investigated the magnetohydrodynamic peristaltic flow of a non-Newtonian fluid in the porous space of a microchannel. They observed that the fluid becomes less prone to nonlinear effects as the magnetic parameter is increased. Bhatti *et al*.^30^ studied the influence of a variable magnetic field on peristalsis in a nonuniform conduit for a non-Newtonian fluid; they concluded that the magnetic parameter influences the flow rate. In dermatology, magnetic NPs are used to obtain high-layout noninvasive nanoimages in dermoscopy, enabling sophisticated diagnosis and treatment for skin disorders^26^. Rathod and Sanjeevkumar^31^ studied the magnetohydrodynamic peristaltic flow of a Newtonian nanofluid in an asymmetric channel. They considered a uniform magnetic field in a direction normal to the flow field and the nanofluid was assumed to be weakly electrically conductive. The authors concluded that a greater magnetic parameter reduces the flow near the centre line, unlike the behaviour of the flow near the boundaries. Mahmoudi and Hadjipanayis^32^ investigated the application of magnetic NPs (MNPs) as a method for treating glioblastoma that are resistant to radiotherapy and chemotherapy. The authors reported that thermotherapy based on MNPs and an alternating magnetic field can be effectively utilized to treat brain tumours. Nealon *et al*.^33^ studied the magnetism of AuNPs and observed that the thermal dependence of their magnetism is weak.

It is widely known that physiological fluids such as blood generally behave as non-Newtonian fluids. Kumar *et al*.^34^ investigated the blood compatibility of AuNPs and their biomedical science applications. Ellahi *et al*.^35^ investigated a blood-based nanofluid in a composite stenosed artery, considering the heat and mass transfer. They further solved the system using the homotopy perturbation technique and observed that the stenosis height enhances the altitude of the impedance profile. Khurshid *et al*.^36^ examined blood clotting for synthesized MNPs and found that binding arises between coagulated particles in a blood clot and that the relaxation time for an individual particle increases. Mekheimer and Elmaboud^37^ investigated blood clotting for a micropolar fluid model through peristalsis of an annulus. Preliminary results showed that at the maximum clot altitude, the pressure rise is enhanced. Finally, Akbar *et al*.^38^ studied NPs of various shapes in a nonuniform channel. A comparison was made among brick, cylinder, and platelet shapes; it was found that the thermal conductivity for the platelet shape structure is higher than that of the brick and cylindrical shapes.

Motivated by the above investigations, our aim is to study AuNPs using the blood-based Carreau fluid model with peristalsis through an annulus. The NPs are expected to exhibit a temperature increase with novel consequences. Multiple factors are investigated in the model, such as an external variable magnetic field and platelet-shaped NPs. The current research may aid in applications for clinical pathology and in the production of science-based cosmetics. Precisely engineered NPs, platelets hither, enable rapid healing in skin conditions due to their natural ability to form a ‘border’ for vascular walls and their reaction with injured tissue based on their flexible shape and complex exterior interactions^39^. These properties render NPs efficient in transporting water-immiscible active ingredients, such as retinoids, by deeply penetrating the skin^40^. Solutions have been derived using the homotopy perturbation method for velocity profiles, friction forces, pressure, and temperature, and quantitative results are presented.

## Mathematical Modelling

**Figure 1:**
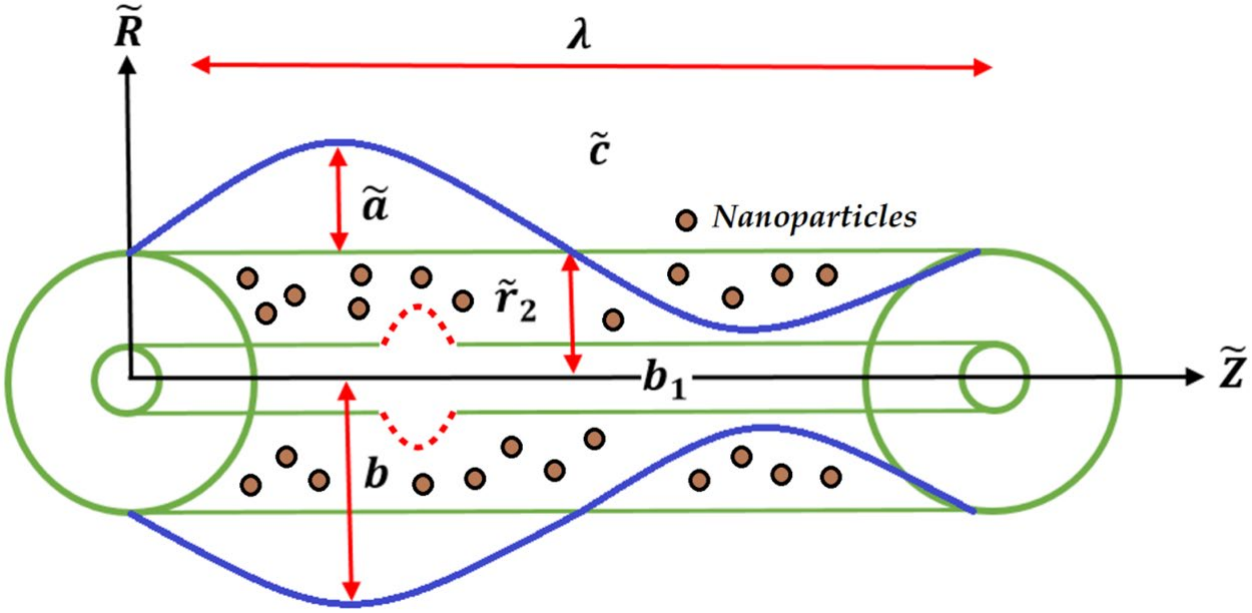
Geometry of the flow problem.

Let us assume two coaxial infinite tubes and a gap between the tubes filled with an electrically conducting, incompressible, Carreau nanofluid with constant density and irrotational properties, comprised of small NPs with a varying magnetic field. The inner tube of the porous annulus is rigid. The outer tube is uniform, with a sinusoidal (or “peristaltic”) wave propagating down its wall at constant velocity $\tilde{{\rm{c}}}$. A cylindrical coordinate system $(\tilde{R},\,\tilde{Z})$ is used to express the problem, where $\tilde{{\rm{Z}}}$ is directed along the centre of the outer and inner tubes and $\tilde{{\rm{R}}}$ points along the radial direction, as displayed in Fig. 1.

The geometry of the wall surface is defined as

1 $${\tilde{r}}_{1}={\tilde{r}}_{0}({b}_{1}+f(\tilde{Z},\tilde{t}))\,{\rm{and}}\,{\tilde{r}}_{2}=b+\tilde{a}\,\sin \,\frac{2\pi }{\lambda }(\tilde{Z}-\tilde{c}\tilde{t}).$$

where *t*, *a*, *b*, *b*_1_, and *λ* are the time, wave amplitude, outer tube radius, inner tube radius, and wavelength, respectively.

The governing equations of motion, concentration, temperature and continuity are expressed as

2 $$-\frac{\partial (\tilde{R}\tilde{U})}{\partial \tilde{R}}=\tilde{R}\frac{\partial \tilde{W}}{\partial \tilde{Z}},$$

3 $$\frac{\partial \tilde{P}}{\partial \tilde{R}}+{\rho }_{nf}(\frac{\partial \tilde{U}}{\partial \tilde{t}}+\tilde{U}\frac{\partial \tilde{U}}{\partial \tilde{R}}+\tilde{W}\frac{\partial \tilde{U}}{\partial \tilde{Z}})=\frac{\partial {S}_{\tilde{R}\tilde{Z}}}{\partial \tilde{Z}}+\frac{1}{\tilde{R}}[\frac{\partial (\tilde{R}{S}_{\tilde{R}\tilde{R}})}{\partial \tilde{R}}-{S}_{\tilde{\theta }\tilde{\theta }}],$$

4 $$\frac{\partial \tilde{P}}{\partial \tilde{Z}}+{\rho }_{nf}(\frac{\partial \tilde{W}}{\partial \tilde{t}}+\tilde{W}\frac{\partial \tilde{W}}{\partial \tilde{Z}}+\tilde{U}\frac{\partial \tilde{W}}{\partial \tilde{R}})=\frac{1}{\tilde{R}}\frac{\partial (\tilde{R}{S}_{\tilde{R}\tilde{Z}})}{\partial \tilde{R}}+\frac{\partial {S}_{\tilde{Z}\tilde{Z}}}{\partial \tilde{Z}}-[{\sigma }_{nf}{\bar{B}}_{0}^{2}(\tilde{R})]\tilde{W}-{(\rho \beta )}_{nf}({\tilde{T}}_{0}-\tilde{T})g,$$

5 $${(\rho {c}_{p})}_{nf}(\frac{\partial \tilde{T}}{\partial \tilde{t}}+\tilde{U}\frac{\partial \tilde{T}}{\partial \tilde{R}}+\tilde{W}\frac{\partial \tilde{T}}{\partial \tilde{Z}})={\tilde{k}}_{nf}(\frac{{\partial }^{2}\tilde{T}}{\partial {\tilde{R}}^{2}}+\frac{1}{\tilde{R}}\frac{\partial \tilde{T}}{\partial \tilde{R}}+\frac{{\partial }^{2}\tilde{T}}{\partial {\tilde{Z}}^{2}}\,)+[{\sigma }_{nf}{\bar{B}}_{0}^{2}(\tilde{R})]{\tilde{W}}^{2}+{S}_{\tilde{R}\tilde{Z}}(\frac{\partial \tilde{W}}{\partial \tilde{R}}),$$

The stress tensor for the Carreau fluid is defined as

6 $${\bf{S}}={\mu }_{nf}{[1+{(\tau \mathop{\gamma }\limits^{\bullet })}^{2}]}^{\frac{n-1}{2}}\mathop{\gamma }\limits^{\bullet }.$$

where

7 $$\begin{array}{c}{\rho }_{nf}=(1-\psi ){\rho }_{f}+\psi \,{\rho }_{f},\,\,\,{\mu }_{nf}=\frac{{\mu }_{f}}{{(1-\bar{\varphi })}^{2.5}},\,{\beta }_{nf}=(1-\psi ){\beta }_{f}+\psi \,{\beta }_{np},\\ {k}_{nf}=\frac{{k}_{p}+(k+1){k}_{f}-(k+1)\psi ({k}_{f}-{k}_{p})}{{k}_{p}+(k+1){k}_{f}+\psi ({k}_{f}-{k}_{p})}{k}_{f},\,\frac{{\sigma }_{nf}}{{\sigma }_{f}}=1+\frac{3(\bar{\gamma }-1)\bar{\varphi }}{(\bar{\gamma }+2)-\bar{\gamma }(\bar{\gamma }-1)\bar{\varphi }},\\ \,\,\,\bar{\gamma }=\frac{{\sigma }_{s}}{{\sigma }_{f}}.\end{array}\}$$

where *ρ*_*nf*_, *μ*_*nf*_, (*ρc*_*p*_)_*nf*_, $\bar{\varphi }$, *σ*_*f*_, *σ*_*s*_, *k*, *ψ*, *k*_*p*_, and *k*_*f*_ are the nanofluid density, dynamic viscosity, nanofluid heat capacitance, NP volume fraction, base fluid electrical conductivity, NP electrical conductivity, shape factor, particle volume fraction, and the conductivities of the particle material and base fluid, respectively.

The thermophysical properties of blood and AuNPs are described in Table 1.

**Table 1:** Thermophysical properties of AuNPs and blood.

Physical properties	${{\boldsymbol{C}}}_{{\boldsymbol{p}}}({\boldsymbol{J}}/{\boldsymbol{kg}}.{\boldsymbol{K}})$	${\boldsymbol{\rho }}({\boldsymbol{kg}}/{{\boldsymbol{m}}}^{3})$	${\boldsymbol{\kappa }}({\boldsymbol{W}}/{\boldsymbol{mK}})$
Blood	3594	1063	0.492
Gold	129	19300	318

To move from a fixed frame to a wave frame, we define the variables $\tilde{Z}=\bar{z}+\tilde{c}\tilde{t},\,\bar{r}=\tilde{R},\tilde{W}=\tilde{c}+\bar{w},$ and $\tilde{U}=\bar{u}.$ We also define the following nondimensional quantities:

8 $$\begin{array}{c}r=\frac{\bar{r}}{b},\,z=\frac{\bar{z}}{\lambda },\,u=\frac{\lambda \bar{u}}{b\tilde{c}},\,w=\frac{\bar{w}}{\tilde{c}},\,\delta =\frac{b}{\lambda },\,\theta =\frac{\tilde{T}-{\tilde{T}}_{1}}{{\tilde{T}}_{0}-{\tilde{T}}_{1}},\,\varphi =\frac{\tilde{a}}{b},\,{r}_{2}=\frac{{\tilde{r}}_{2}}{\tilde{b}},\\ {M}^{2}=\frac{{\sigma }_{f}{B}_{0}^{2}(r){b}^{2}}{{\mu }_{f}},\,{G}_{r}=\frac{g{\tilde{b}}^{2}({\tilde{T}}_{0}-{\tilde{T}}_{1}){\rho }_{f}{\beta }_{f}}{{\mu }_{f}\tilde{c}},\,{B}_{r}={P}_{r}{E}_{c},\,t=\frac{\tilde{c}\tilde{t}}{\lambda },\,k=\frac{{k}_{1}}{{b}^{2}},\\ {\rm{Re}}=\frac{{\rho }_{f}b\tilde{c}}{{\mu }_{f}},\,{P}_{r}=\frac{{(\rho {c}_{p})}_{f}{\nu }_{f}}{{k}_{f}},\,{E}_{c}=\frac{{\tilde{c}}^{2}}{({\tilde{T}}_{0}-{\tilde{T}}_{1}){c}_{p}}.\end{array}\}$$

where *E*_*c*_, *P*_*r*_, *G*_*r*_, *B*_*r*_, *M*, *k*_*f*_, *k*_*p*_, *B*_0_, ${\tilde{k}}_{nf}$, Re, $\tilde{T}$, *ϕ*, and *δ* are the Eckert number, Prandtl number, thermal Grashof number, Brinkman number, Hartmann number, nanofluid thermal conductivity, NP thermal conductivity, applied magnetic field, effective thermal conductivity, Reynolds number, temperature, amplitude ratio, and wave number, respectively.

Substituting Eq. (9) into Eqs (1–6) using the long wavelength approximation and a creeping flow regime yields

9 $$\frac{\partial p}{\partial r}=0,$$

10 $$\frac{\partial p}{\partial z}=\frac{1}{r{B}_{3}}\frac{\partial }{\partial r}[r\{\frac{\partial w}{\partial r}+\frac{1}{2}W{e}^{2}(n-1){(\frac{\partial w}{\partial r})}^{3}\}]+{G}_{r}{B}_{1}\theta -{B}_{2}{M}^{2}(r)(w+1),$$

11 $${B}_{4}\frac{1}{r}\frac{\partial }{\partial r}(r\frac{\partial \theta }{\partial r})+{B}_{r}{B}_{2}{M}^{2}(r){(w+1)}^{2}+\frac{{B}_{r}}{{B}_{3}}\{{(\frac{\partial w}{\partial r})}^{2}+\frac{1}{2}W{e}^{2}(n-1){(\frac{\partial w}{\partial r})}^{4}\}=0.$$

where

12 $$\begin{array}{c}{B}_{1}=1-\psi +\psi (\frac{{(\rho \beta )}_{nf}}{{(\rho \beta )}_{f}}),\,{B}_{2}=1+\frac{3(\bar{\gamma }-1)\psi }{(\bar{\gamma }+2)-\bar{\gamma }(\bar{\gamma }-1)\psi },\,{B}_{r}={P}_{r}{E}_{c}\\ {B}_{3}={(1-\psi )}^{2.5},\,{B}_{4}=\frac{{k}_{p}+(k-1){k}_{f}-(k-1)\psi ({k}_{f}-{k}_{p})}{{k}_{p}+(k-1){k}_{f}+\bar{\varphi }({k}_{f}-{k}_{p})}.\end{array}\}$$

subject to the following boundary conditions:

13 $$w=-\,1,\,\theta =1,\,{\rm{at}}\,r={r}_{1}=\dot{o}+{\rm{\Gamma }}{e}^{-\pi {[z-zd-0.5]}^{2}}\,{\rm{and}}\,w=-\,1,\,\theta =0,\,{\rm{at}}\,r={r}_{2}=1+\varphi \,\sin \,2\pi z.\,$$

Γ, *θ*, *We* and *n* are the clot height, temperature, Weissenberg number, and power law index, respectively.

## Series Solutions of the Problem

The solutions of Eqs (9–11) can be determined using a homotopy perturbation scheme. The homotopy for Eqs (9–11) is defined as

14 $$h({\rm{\Theta }},\tilde{q})=(1-\tilde{q})[L({\rm{\Theta }})-L({\tilde{{\rm{\Theta }}}}_{0})]+\tilde{q}[\begin{array}{c}L({\rm{\Theta }})+\frac{W{e}^{2}(n-1)}{2r}{(\frac{\partial {\rm{\Theta }}}{\partial r})}^{3}+\frac{3W{e}^{2}(n-1)}{2}\frac{{\partial }^{2}{\rm{\Theta }}}{\partial {r}^{2}}{(\frac{\partial {\rm{\Theta }}}{\partial r})}^{2}\\ +\,{G}_{r}{B}_{3}{B}_{1}\vartheta -{B}_{2}{B}_{3}{M}^{2}(r)({\rm{\Theta }}+1)-{B}_{3}\frac{\partial p}{\partial z}+\frac{1}{r}\frac{\partial {\rm{\Theta }}}{\partial r}\end{array}],$$

15 $$h(\vartheta ,\tilde{q})=(1-\tilde{q})[L(\vartheta )-L({\bar{\vartheta }}_{0})]+\tilde{q}[\begin{array}{c}L(\vartheta )+\frac{{B}_{r}}{{B}_{4}}{B}_{2}{M}^{2}(r){({\rm{\Theta }}+1)}^{2}+\frac{1}{r}\frac{\partial \vartheta }{\partial r}\\ +\,\frac{{B}_{r}}{{B}_{4}{B}_{3}}[{(\frac{\partial {\rm{\Theta }}}{\partial r})}^{2}+\frac{W{e}^{2}(n-1)}{2}{(\frac{\partial {\rm{\Theta }}}{\partial r})}^{4}]\end{array}],$$

where $\tilde{q}$ is an embedding parameter. The linear operator *L* is taken in the following form:

16 $$L=\frac{{\partial }^{2}}{\partial \,{r}^{2}},$$

and the initial expressions for the above linear operators are defined as

17 $${\tilde{{\rm{\Theta }}}}_{0}=-\,1+(r-{r}_{1})(r-{r}_{2}),\,{\bar{\vartheta }}_{0}=\frac{r-{r}_{2}}{{r}_{1}-{r}_{2}},$$

The expression for the variable magnetic field is defined as^41^

18 $$M(r)=\frac{M}{r}.$$

The following expansions are employed:

19 $${\rm{\Theta }}(r)={{\rm{\Theta }}}_{0}(r)+\tilde{q}\,{{\rm{\Theta }}}_{1}(r)+{\tilde{q}}^{2}{{\rm{\Theta }}}_{2}(r)+\ldots ,$$

20 $$\vartheta (r)={\vartheta }_{0}(r)+\tilde{q}{\vartheta }_{1}(r)+{\tilde{q}}^{2}{\vartheta }_{2}(r)+\ldots .,$$

By applying the series expansion in Eqs (19 and 20) to the homotopy sets represented by Eqs (14 and 15) and comparing equal powers of $\tilde{q}$, a system of linear differential equations can be obtained. Using the property of the homotopy perturbation scheme, i.e., $\tilde{q}\to 1$, we have

21 $$w(r)={\rm{\Theta }}(r)={{\rm{\Theta }}}_{0}(r)+{{\rm{\Theta }}}_{1}(r)+{{\rm{\Theta }}}_{2}(r)+\ldots ,$$

22 $$\theta (r)=\vartheta (r)={\vartheta }_{0}(r)+{\vartheta }_{1}(r)+{\vartheta }_{2}(r)+\ldots ,$$

The final solution form for the velocity and temperature profiles can then be written in a simplified form as

23 $$w(r)={w}_{0}+{w}_{1}r+{w}_{2}{r}^{2}+{w}_{3}{r}^{3}+{w}_{4}{r}^{4},$$

24 $$\theta (r)={\theta }_{0}+{\theta }_{1}\,\mathrm{log}\,r+{\theta }_{2}r+{\theta }_{3}r\,\mathrm{log}\,r+{\theta }_{4}{r}^{2}+{\theta }_{5}{r}^{3}+{\theta }_{6}{r}^{4}+{\theta }_{7}{r}^{5}+{\theta }_{8}{r}^{6}.$$

The constant in the above equations can be found using routine calculations. The instantaneous volume flow rate *Q* can be calculated as^42^

25 $$Q={\int }_{{r}_{1}}^{{r}_{2}}2rw\,(r,\,z){\rm{d}}r.$$

The mean flow rate over the period of one wavelength can be written as

26 $$\bar{Q}=Q+\frac{{\varphi }^{2}}{2}+1-{\dot{U}}^{2}.$$

where $\dot{U}$ is the inner tube radius.

The pressure rise Δ*p* and the friction forces for the outer tube *F*_*o*_ and inner tube *F*_*i*_ over the period of one wavelength can be written as

27 $$\begin{array}{rcl}{\rm{\Delta }}p & = & {\int }_{0}^{1}\,\frac{dp}{dz}{\rm{d}}z,\\ {F}_{i} & = & {\int }_{0}^{1}\,(\,\,-\,{r}_{1}^{2})\frac{dp}{dz}{\rm{d}}z,\\ {F}_{o} & = & {\int }_{0}^{1}\,(\,\,-\,{r}_{2}^{2})\frac{dp}{dz}{\rm{d}}z.\end{array}\}$$

## Results and Discussion

The purpose of this section is to determine the influence of the pertinent parameters on physical variables in the current NP blood flow model. Mathematica was utilized to deduce the physical influence of the inner tube radius *ϵ*, maximum clot height Γ, power law index *n*, Hartmann number *M*, Weissenberg number *We*, particle volume fraction *ψ*, volume flow rate $\bar{Q}$, thermal Grashof number *G*_*r*_, and Brinkman number *B*_*r*_ on the distributions of velocity *w*, temperature *θ*, pressure rise Δ*p*, pressure gradient d*p*/d*z*, friction forces on the inner and outer tubes (*F*_*i*_ and *F*_*o*_), and contoured streamlines. Since the problem involves many nondimensional parameters, for the sake of conciseness, in the ongoing discussion, the parameters are set as (*ϵ*, $\bar{Q}$, *n*, *B*_*r*_, *ψ*, *G*_*r*_, *We*, Γ, *ϕ*, *M*) = (0.1, 0.8, 5, 3, 0.15, 2, 0.9, 0.1, 0.2, 2), and only one parameter is varied at a time.

**Figure 2:**
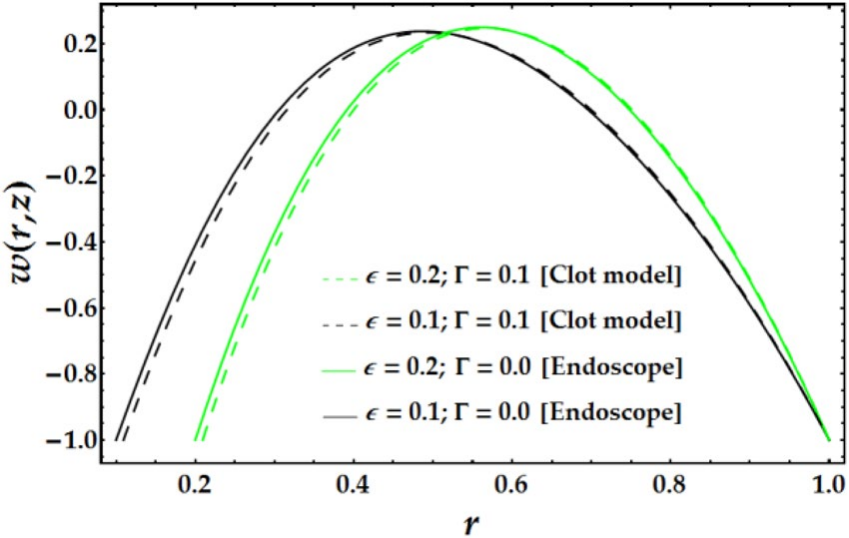
Velocity profile for different values of *ϵ* and Γ.

**Figure 3:**
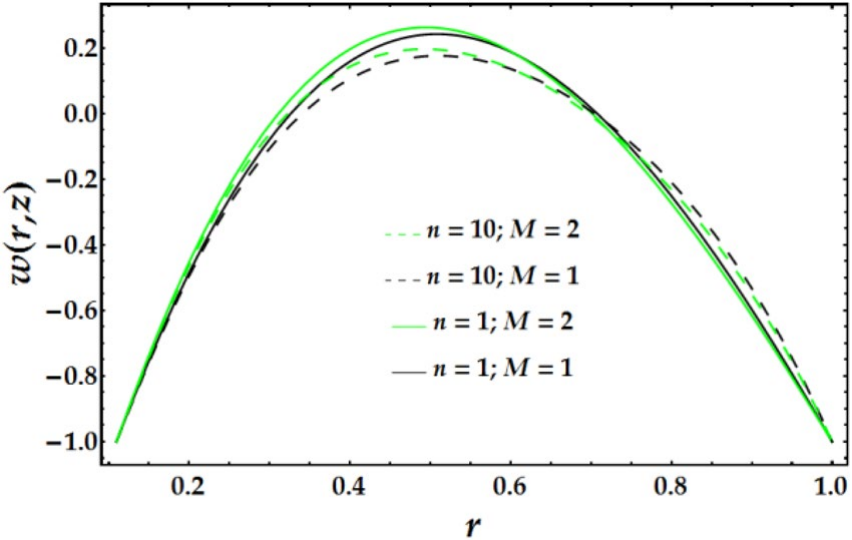
Velocity profile for different values of *n* and *M*.

**Figure 4:**
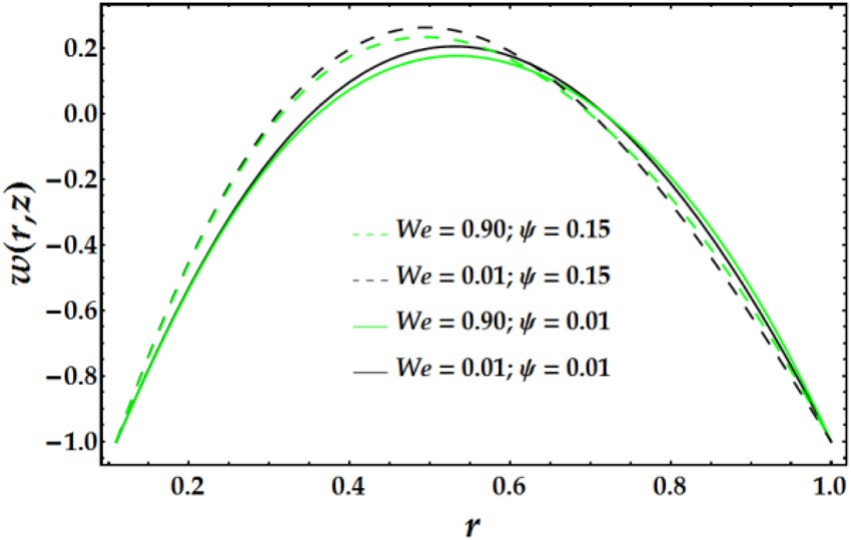
Velocity profile for different values of *We* and *ψ*.

**Figure 5:**
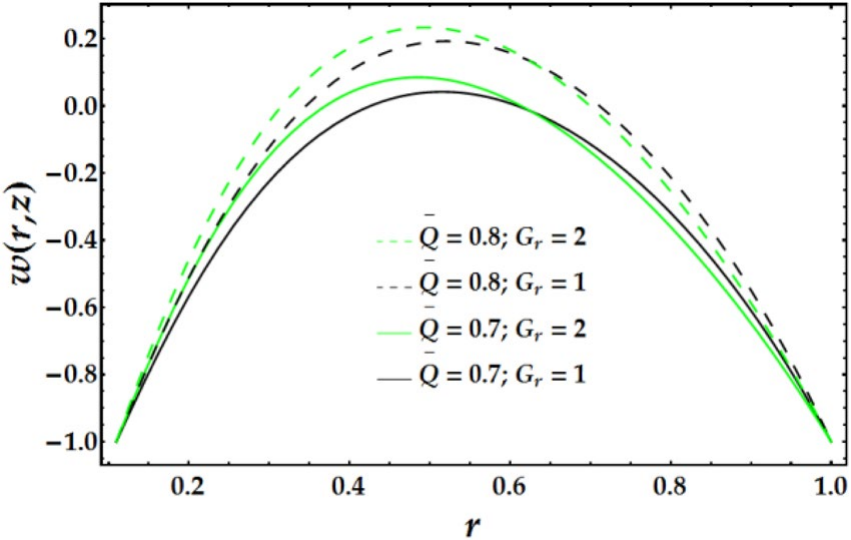
Velocity profile for different values of $\overline{Q}$ and *G*_*r*_.

In Figs 2–5, the two-dimensional velocity profile is plotted versus the radial coordinate r for various values of the relevant parameters. Inspection of Fig. 2 shows that increasing the inner tube radius causes the nanofluid to accelerate until r = 0.57, at which point the behaviour is reversed with an increase in *ϵ*. For a fixed value of *ϵ*, an increasing clot height reduces the nanofluid velocity until *r* = 0.57; increases beyond this point have only a slight effect. The maximum velocity occurs near the centre of the annulus. Figure 3 presents the velocity distribution with r for various values of *n* and *M*. Examination of the figure shows that n has a decreasing effect on the nanofluid velocity in three-fourths of the annulus before its impact is reversed. Similarly, the influence of *M* on the velocity profile is identical to that of *n*. The variations in nanofluid velocity with *We* and *ψ* are displayed in Fig. 4. It is observed that the nanofluid flow decelerates with an increase in *We*, whereas an increasing *ψ* accelerates the flow until *r* = 0.65, at which point, the flow shows the opposite behaviour. The influence of $\bar{Q}$ and *G*_*r*_ on the velocity distribution is illustrated in Fig. 5. It is shown that the velocity profile increases substantially with increasing $\bar{Q}$. A different behaviour is seen for increasing *G*_*r*_; the flow increases until r = 0.65 and then decreases.

**Figure 6:**
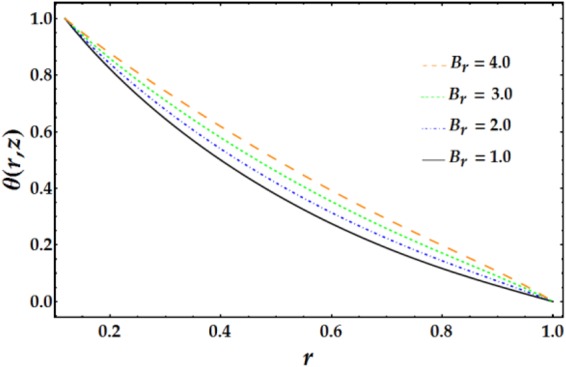
Temperature profile for different values of *B*_*r*_.

**Figure 7:**
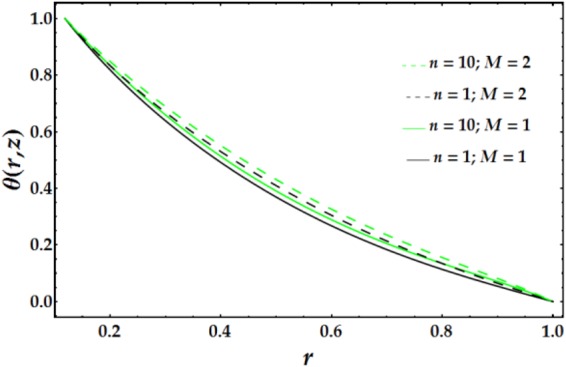
Temperature profile for different values of *n* and *M*.

**Figure 8:**
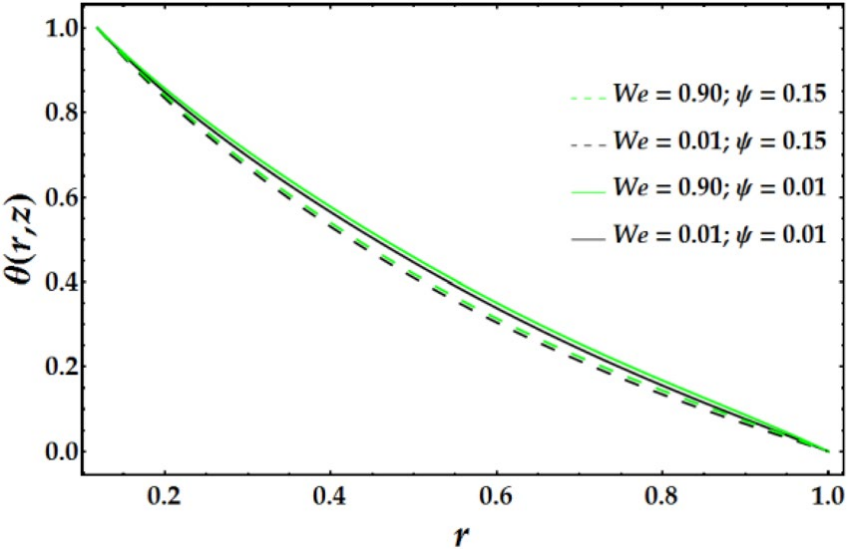
Temperature profile for different values of *We* and *ψ*.

Variations in the nanofluid temperature in the wavy annulus with the radial coordinate *r* for various parameter values are plotted in Figs 6–8. In general, the temperature profile is semi-parabolic, and the values decrease with increasing *r*. Figure 6 demonstrates the influence of *B*_*r*_ on the temperature profile; increasing *B*_*r*_ leads to an increased *θ* when the other parameters are held constant. This behaviour corresponds to typical heat transfer, where the nanofluid’s temperature increases with an increase in the external heating and the heat conduction produced by viscous dissipation occurs at a lower rate, resulting in a higher temperature. Figure 7 depicts the variation of *θ* with *r* for various values of *n* and *M*. It is shown that the temperature increases with increasing n. Ditto the impact of *M* on the temperature distribution. Figure 8 demonstrates the effect of *We* and *ψ* on *θ* for fixed parameter values. It is observed that the temperature increases slightly with increasing *We*, whereas it decreases with increasing *ψ*.

**Figure 9:**
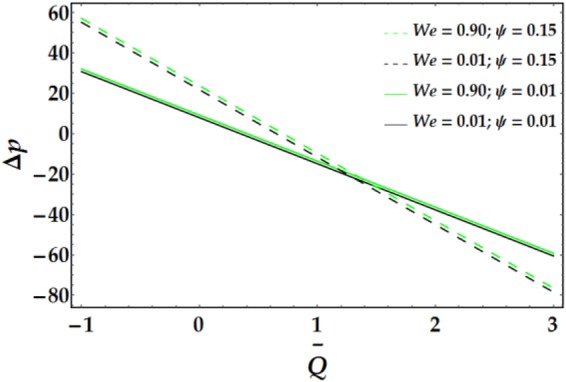
Pressure rise for different values of *We* and *ψ*.

**Figure 10:**
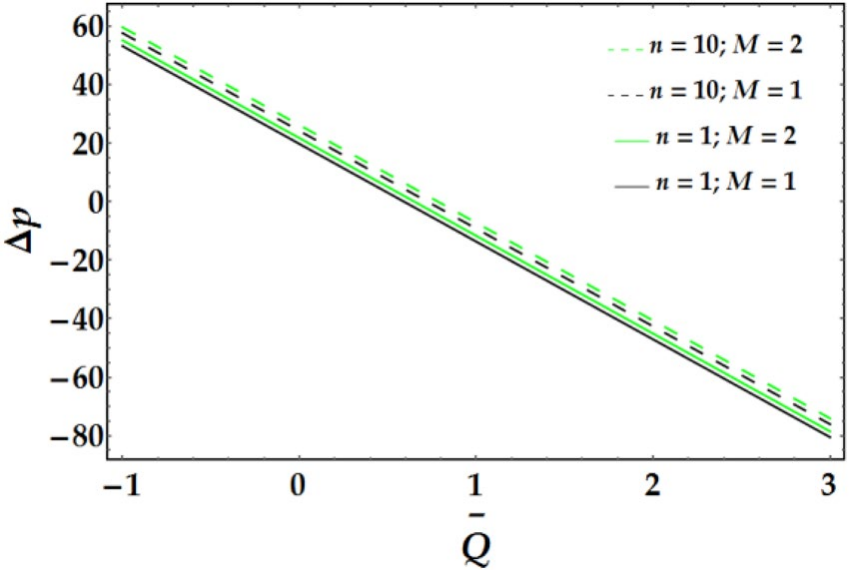
Pressure rise for different values of *n* and *M*.

**Figure 11:**
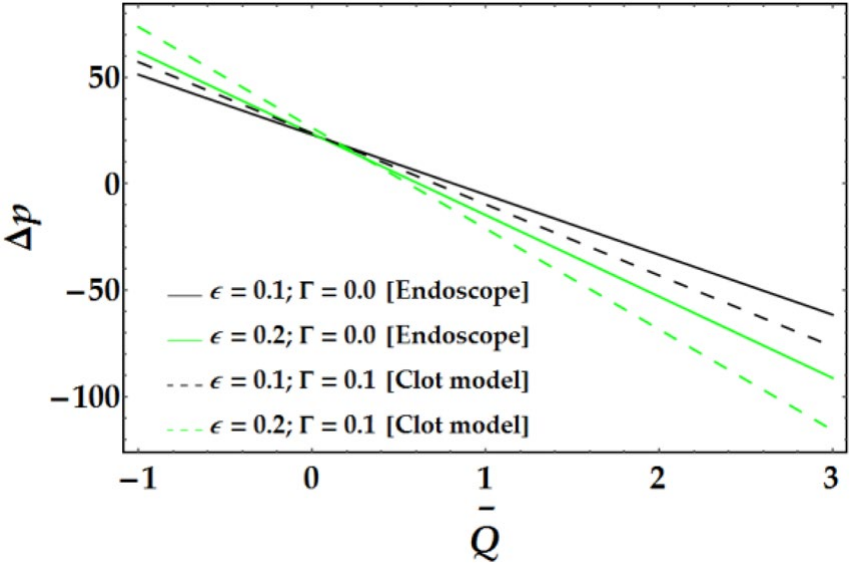
Pressure rise for different values of *ϵ* and Γ.

**Figure 12:**
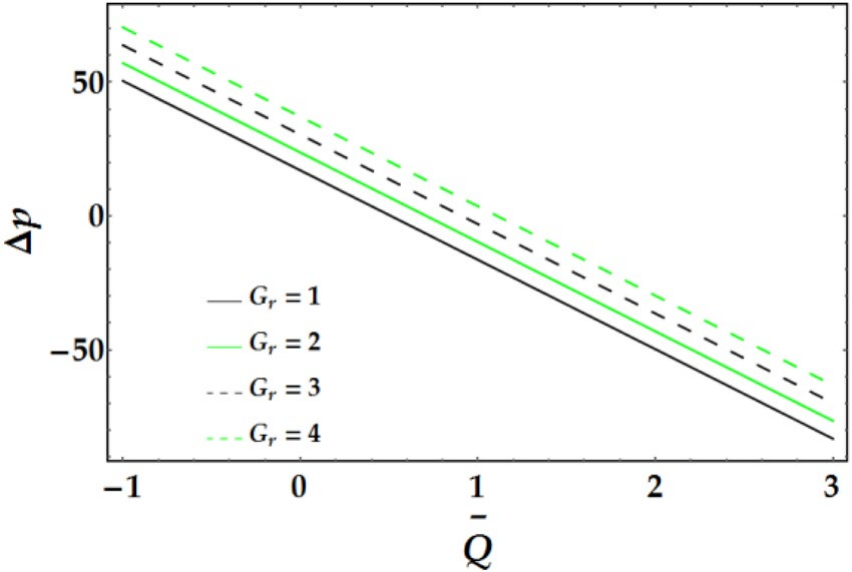
Pressure rise for different values of *G*_*r*_.

Figures 9–12 present the pressure rise versus $\overline{Q}$ for fixed parameter values. Figure 9 elucidates the variation of Δp with $\overline{Q}$ for various values of *We* and *ψ*. It is seen that Δp increases slightly with increasing *We*. Conversely, an increase in *ψ* leads to a pressure rise below a certain value of $\overline{Q}$ (= 1.22); the behaviour of Δp is reversed for higher values of $\overline{Q}.$ The influence of *n* and *M* on the pressure rise is illustrated in Fig. 10; both parameters have a slight increasing effect on Δp for various values of $\overline{Q}$. Figure 11 reveals the endoscopic effect and clot model for Δp versus $\overline{Q}$. It is revealed that when the inner tube radius increases, the pressure rise increases until $\bar{Q}=0.2$, after which the behaviour of Δp is reversed for progressive values of $\overline{Q}$. A similar trend is observed for Γ and Δp at fixed values of *ϵ*. The influence of the Grashof number on the pressure rise is demonstrated in Fig. 12, where it is observed that *G*_*r*_ causes a considerable increase in the pressure rise at fixed parameter values.

**Figure 13:**
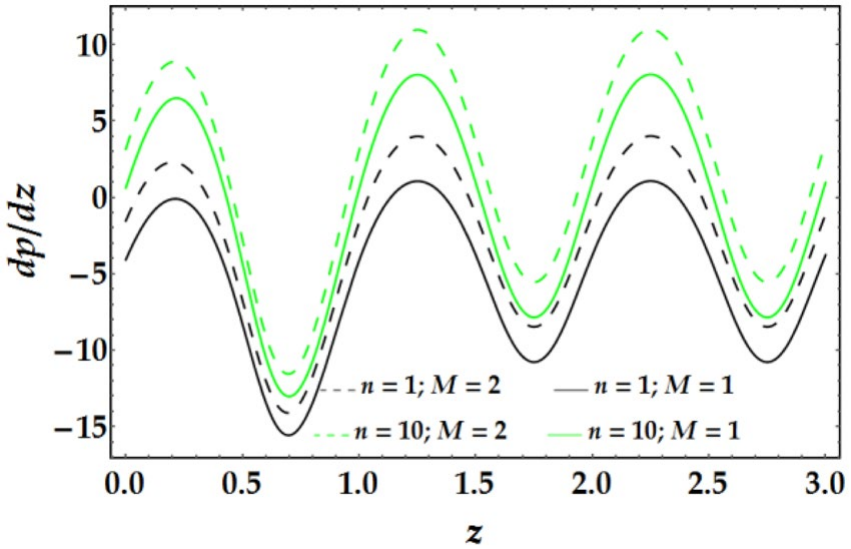
Pressure gradient for different values of *n* and *M*.

**Figure 14:**
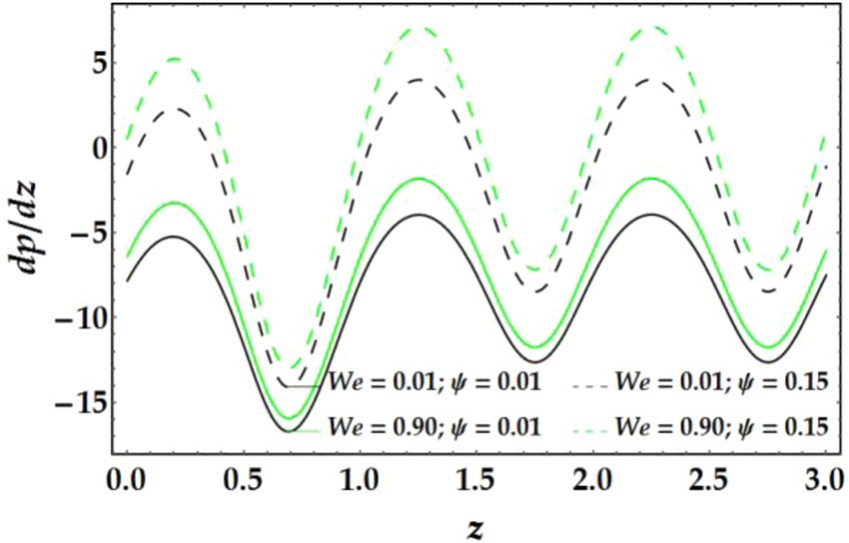
Pressure gradient for different values of *We* and *ψ*.

**Figure 15:**
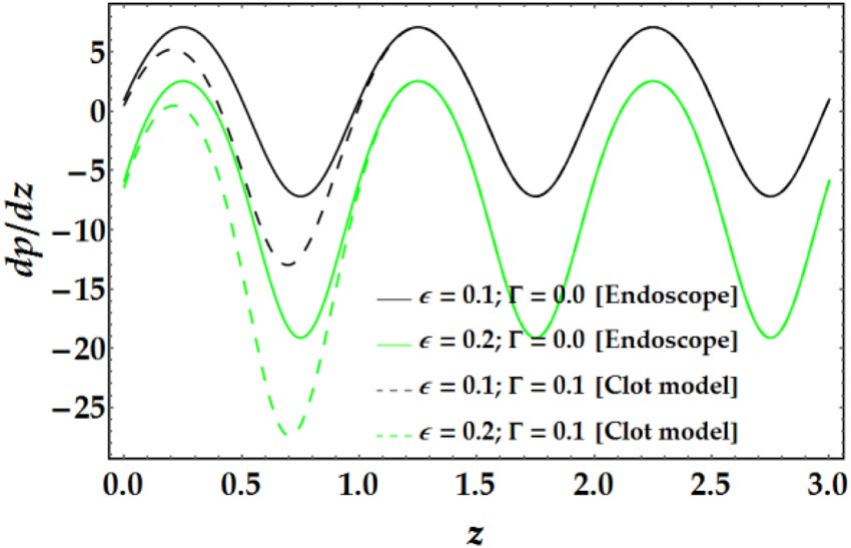
Pressure gradient for different values of *ϵ* and Γ.

**Figure 16:**
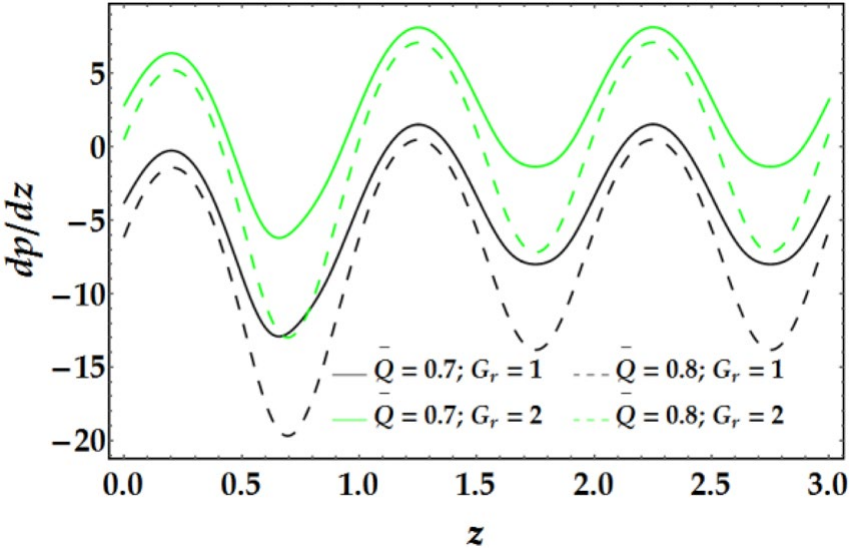
Pressure gradient for different values of $\bar{Q}$ and *G*_*r*_.

The influences of *n*, *M*, *We*, *ψ*, *ϵ*, Γ, .., and *G*_*r*_ on the pressure gradient versus *z* are illustrated in Figs 13–16. Despite the increase in pressure gradient due to the inner tube radius, the impact of *ϵ* coincides with higher values of clot altitude at *z* > 1, as shown in Fig. 15. It is observed that *n*, *M*, *We*, *ψ*, and *G*_*r*_ cause a rapid increase in the pressure gradient while *ϵ*, Γ and $\overline{Q}$ have the opposite impact on *dp*/*dz* when the other parameters are held constant.

**Figure 17:**
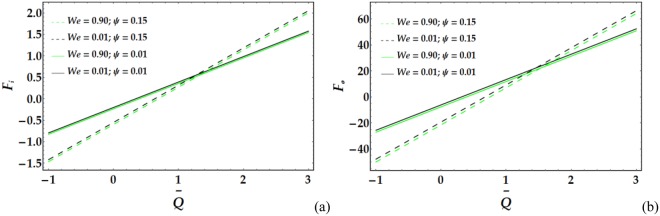
Friction forces for different values of *We* and *ψ* at the (**a**) inner tube and (**b**) outer tube.

**Figure 18:**
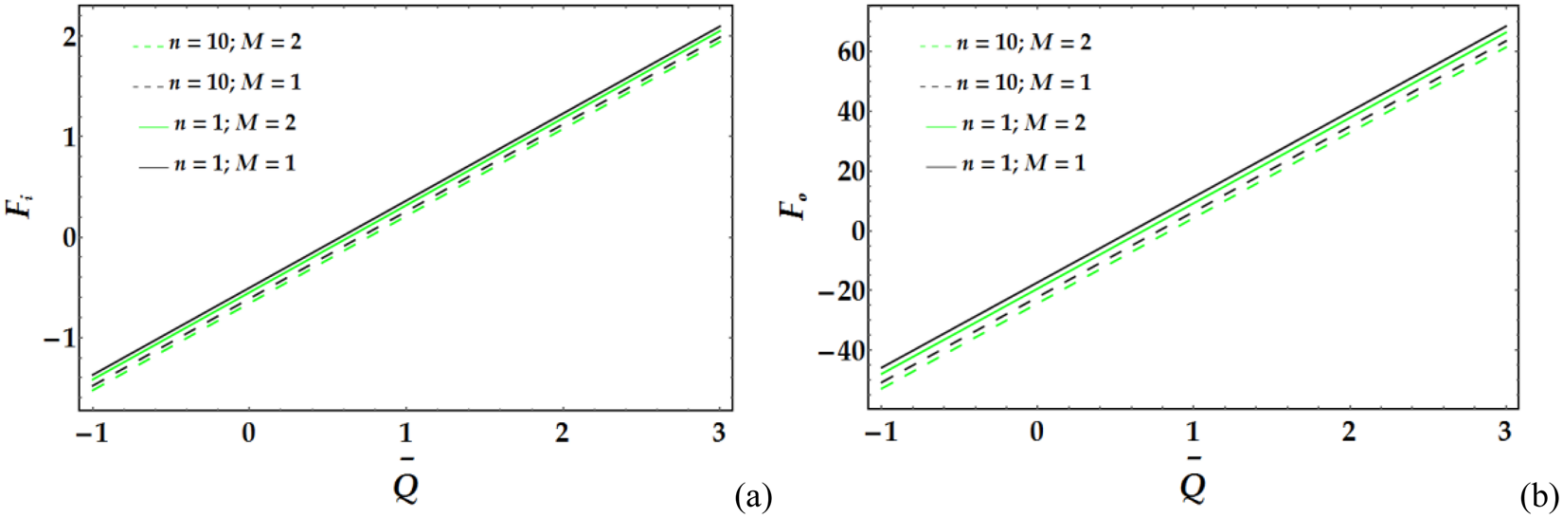
Friction forces for different values of *n* and *M* at the (**a**) inner tube and (**b**) outer tube.

**Figure 19:**
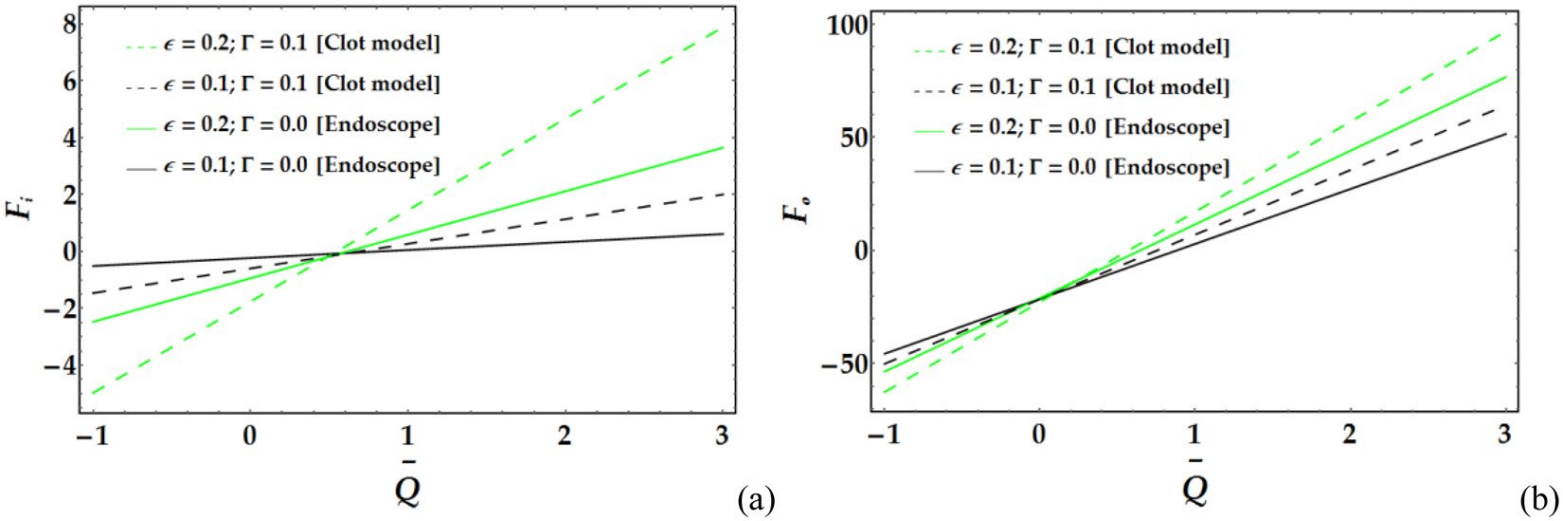
Friction forces for different values of *ϵ* and Γ at the (**a**) inner tube and (**b**) outer tube.

**Figure 20:**
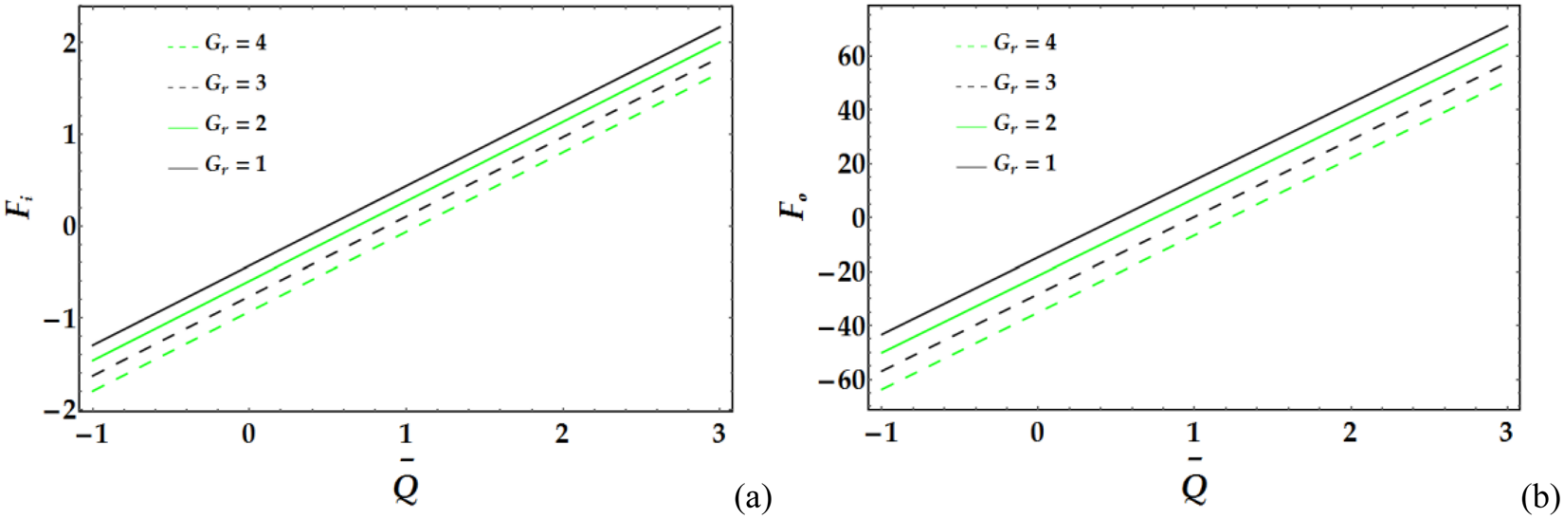
Friction forces for different values of *G*_*r*_ at the (**a**) inner tube and (**b**) outer tube.

Figures 17–20 present the friction forces at the inner and outer tubes for various values of the parameters under consideration. Figure 17 displays the variations in *F*_*i*_ and *F*_*i*_ with $\overline{Q}$ for various values of the considered parameters. It is observed that *F*_*i*_ and *F*_*i*_ are weakly affected by *We*. In addition, *F*_*i*_ and *F*_*i*_ rapidly decrease with increasing *ψ* until a certain value of $\overline{Q}$ (=1.3) is reached, at which point they begin to increase. Figure 18 depicts the friction forces with varying *M* and *n*; both parameters cause an increase in *F*_*i*_ and *F*_*i*_ for fixed values of the pertinent parameters. Figure 19 demonstrates the impact of *ϵ* and Γ on the friction forces versus $\overline{Q}$. A considerable decrease in *F*_*i*_ and *F*_*i*_ occurs for increasing *ϵ* and Γ until $\overline{Q}$ = 0.58 and 0.1 at the inner and outer tube, respectively, beyond which the friction forces increase. Figure 20 reveals the behaviour of *F*_*i*_ and *F*_*o*_ for various values of the Grashof number; increasing *G*_*r*_ causes a rapid decrease in the friction forces for fixed values of the parameters under consideration.

**Figure 21:**
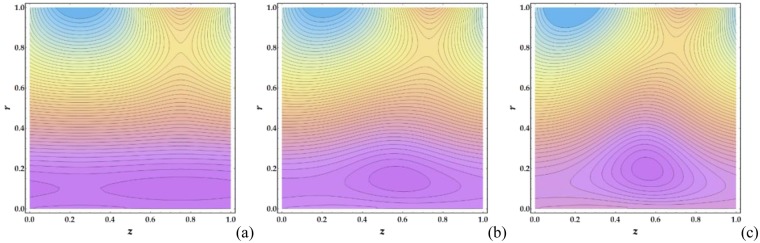
Streamlines for different values of Γ (**a**) 0, (**b**) 0.05, (**c**) 0.1.

**Figure 22:**
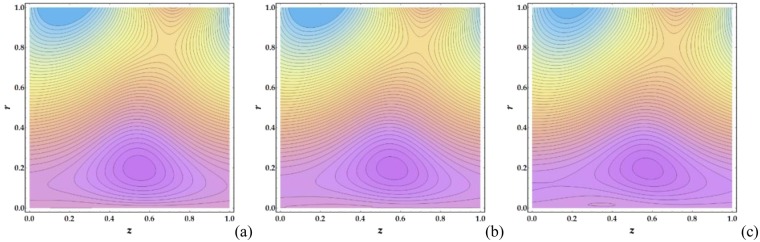
Streamlines for different values of *M* (**a**) 1, (**b**) 2, (**c**) 3.

**Figure 23:**
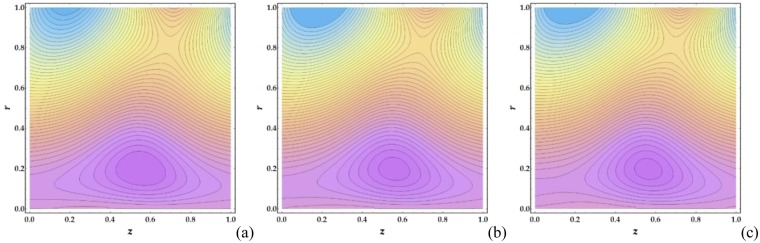
Streamlines for different values of *n*: (**a**) 1, (**b**) 5, (**c**) 10.

**Figure 24:**
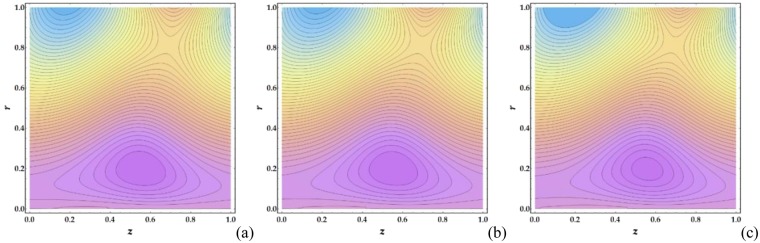
Streamlines for different values of *We* (**a**) 0.01, (**b**) 0.5, (**c**) 0.9.

Figures 21–24 illustrate the streamlines and trapping phenomenon that occur in a moving frame with circulating fluid. It is observed from Fig. 21 that the clot altitude does not have a significant effect on the number of trapping streamlines despite a significant increase in the number of trapped boluses. Figure 22 shows that increasing *M* leads to a significant decrease in the number of trapped boluses. It is shown in Fig. 23 that there is a slight increase in the number of trapped boluses with increasing *n*, whereas the number of enclosed streamlines remains nearly constant. Finally, Fig. 24 shows that as *We* is increased, the number of contouring nanofluid streamlines increases slightly with a decrease in the width of the trapped boluses.

## Concluding Remarks

In this work, the peristaltic propulsion of a blood model with AuNPs through an annulus is theoretically investigated. Various theories were investigated to explain the unexpected impact of a variable magnetic field on platelet-shaped NPs, which have a higher thermal conductivity than brick or cylindrical shaped NPs. Multiple factors were discussed, including the clot altitude and the inner tube radius, which have a direct influence on the nanofluid flow model. The resulting highly nonlinear differential equations were simplified under a lubrication approach and were further resolved using a homotopy perturbation technique. The distributions of velocity, temperature, pressure rise, pressure gradient, friction forces, and enclosing streamlines were calculated and presented. The main conclusions are as follows:

(i). Despite the decreasing effect of *ϵ* on the pressure gradient, this parameter has a temporary decreasing/increasing effect on the friction forces/nanofluid velocity below a certain value, at which point, the behaviour is reversed.
(ii). Despite the reduction in the pressure gradient, velocity distribution and friction forces caused by the clot altitude until a certain value is reached, an increase in clot altitude increases the number of trapping streamlines of the nanofluid.
(iii). The maximum velocity occurs at the centre of the annulus.
(iv). Although increasing *n* reduces the nanofluid velocity in three-fourths of the annulus, it enhances the temperature, pressure gradient, and the number of trapped boluses in the nanofluid. *M* has a similar effect on the aforementioned physical variables, except for the streamline number.
(v). The friction forces at the inner and outer tubes decrease with increasing *We*, whereas the pressure gradient increases with increasing *We*.
(vi). An increased particle volume fraction increases the velocity profile and pressure rise below a certain value.
(vii). Unlike the behaviour of the pressure gradient with $\bar{Q}$, the nanofluid velocity increases rapidly with $\bar{Q}$.
(viii). Unlike the impact of *G*_*r*_ on the pressure rise, it substantially decreases the friction forces.
